# INTERSTAARS: Attention training for infants with elevated likelihood of developing ADHD: A proof-of-concept randomised controlled trial

**DOI:** 10.1038/s41398-021-01698-9

**Published:** 2021-12-20

**Authors:** Amy Goodwin, Emily J. H. Jones, Simona Salomone, Luke Mason, Rebecca Holman, Jannath Begum-Ali, Anna Hunt, Martin Ruddock, George Vamvakas, Emily Robinson, Catherine J. Holden, Chloë Taylor, Tim J. Smith, Edmund Sonuga-Barke, Patrick Bolton, Tony Charman, Andrew Pickles, Sam Wass, Mark H. Johnson, and the INTERSTAARS team

**Affiliations:** 1 Institute of Psychiatry, Psychology & Neuroscience, King’s College London, London, UK; 2 Centre for Brain and Cognitive Development, Department of Psychology, Birkbeck College, University of London, London, UK; 3 Centre for Oral Clinical Research (COCR) & Centre for Immunobiology and Regenerative Medicine, Institute of Dentistry, Barts and The London School of Medicine and Dentistry, London, UK; 4 Department of Psychology, University of Southampton, Southampton, UK; 5 Department of Psychology, University of East London, London, UK; 6 Department of Psychology, University of Cambridge, Cambridge, UK

**Keywords:** Psychology, ADHD

## Abstract

Attention-deficit/hyperactivity disorder (ADHD) is first diagnosed during middle childhood, when patterns of difficulty are often established. Pre-emptive approaches that strengthen developing cognitive systems could offer an alternative to post-diagnostic interventions. This proof-of-concept randomised controlled trial (RCT) tested whether computerised gaze-based attention training is feasible and improves attention in infants liable to develop ADHD. Forty-three 9- to 16-month-old infants with a first-degree relative with ADHD were recruited (11/2015–11/2018) at two UK sites and randomised with minimisation by site and sex to receive 9 weekly sessions of either (a) gaze-contingent attention training (intervention; *n* = 20); or (b) infant-friendly passive viewing of videos (control, *n* = 23). Sessions were delivered at home with blinded outcome assessments. The primary outcome was a composite of attention measures jointly analysed via a multivariate ANCOVA with a combined effect size (ES) from coefficients at baseline, midpoint and endpoint (Registration: ISRCTN37683928). Uptake and compliance was good but intention-to-treat analysis showed no significant differences between 20 intervention and 23 control infants on primary (ES −0.4, 95% CI −0.9 to 0.2; Complier-Average-Causal Effect ES −0.6, 95% CI −1.6 to 0.5) or secondary outcomes (behavioural attention). There were no adverse effects on sleep but a small increase in post-intervention session fussiness. Although feasible, there was no support for short-term effects of gaze-based attention training on attention skills in early ADHD. Longer-term outcomes remain to be assessed. The study highlights challenges and opportunities for pre-emptive intervention approaches to the management of ADHD.

## Introduction

Attention-deficit/hyperactivity disorder (ADHD) is a neurodevelopmental disorder characterised by inattention, hyperactivity and impulsivity with an estimated prevalence of around five percent in the general population [1, 2]. ADHD is typically first diagnosed and treated in middle childhood, by which time it is already associated with a broad pattern of difficulties across multiple domains of everyday functioning that usually persists into adulthood [3], and which can impact on quality of life [4]. Medication has efficacy in reducing core ADHD symptoms, although its effects on important domains of functioning is limited [5] and long-term benefits remain uncertain [6, 7]. There is resistance to the use of medication by some parents and professionals [8–10], especially with younger children; recommended parent-training alternatives can reduce conduct problems but do not affect core ADHD symptomatology [11]. Alternative non-pharmacological approaches that target differences in cognitive systems thought to be core to ADHD (e.g., attention and working memory training) have been developed. However, although such approaches might improve performance on tasks similar to those trained, there is little evidence they reduce core ADHD symptoms [12, 13].

Preventative strategies offer an alternative to the non-pharmacological management of ADHD. The benefits of cognitive training could be increased and the likelihood of ADHD reduced through targeting brain systems during periods of increased plasticity in early development. Indeed, there is emerging evidence that ADHD-related atypicalities in attention systems may be present in infancy long before the emergence of the disorder [14]. Could targeting these systems reduce the likelihood of later ADHD? The success of such a strategy depends on (i) identifying vulnerable individuals during early development before ADHD onset is evident and (ii) having a way of delivering cognitive training to very young pre-verbal children. First, ADHD is a strongly familial condition, with a 13-fold increase in ADHD diagnosis in children with a first-degree relative with the condition [15]. This enables the prospective identification of infants with an elevated likelihood of developing ADHD. Second, a recent proof-of-concept study supports the use of non-invasive eye tracking to enable cognitive training protocols in infancy [16]. In this study, typically developing 11-month-old-infants viewed animated games designed to target emerging executive attention skills [16]. The games were designed to be infant-friendly, by using high-contrast colourful animations such as cartoon animals and characters. Eye-tracking (which uses infrared light to track the direction of an infant’s gaze) was used in real time to adaptively change the games contingent on where on screen the infant was looking, thus creating an interactive game, and allowing for the game to become more challenging contingent on the infant’s performance. Cognitive domains targeted by the games included sustained attention (maintaining gaze on a stimulus to receive an audiovisual reward), working memory (remembering where to look to receive an audiovisual reward, e.g. an infant-friendly animation), and cognitive control (inhibiting a previous rule to learn a new one, and receive an audiovisual reward). Following training, infants showed training effects on a battery of cognitive tasks compared to an untrained, active control group. Subsequent studies have replicated key effects [17, 18] and suggest training-related changes in social orienting [17], physiological indices of stress [18, 19] and sustained attention [20]. Taken together, there appears to be great potential for pre-emptive attention training to change trajectories in ADHD. To test this hypothesis, we examine (1) whether gaze-contingent attention training can be feasibly delivered in the context of a double-blinded, active-controlled randomised trial with infants (9- to 16-months) with elevated familial likelihood for ADHD, and (2) whether the training produces significant improvements in immediate attention skills relative to a video-viewing control group.

## Methods

Of note, full methodological details can be found in [21].

### Study design, governance and setting

INTERSTAARS was a blinded Phase 2 randomised controlled trial at 2 sites in the United Kingdom. Ethical approval was obtained from the London Central NHS Research Ethics Service (15/LO/0407). Parents of all participants provided written informed consent. A study Data Monitoring and Ethics Committee (DMEC) of an independent psychiatrist chair, statistician and ethicist was appointed, and convened three times during the setup and course of the trial.

### Participants

**Fig. 1:**
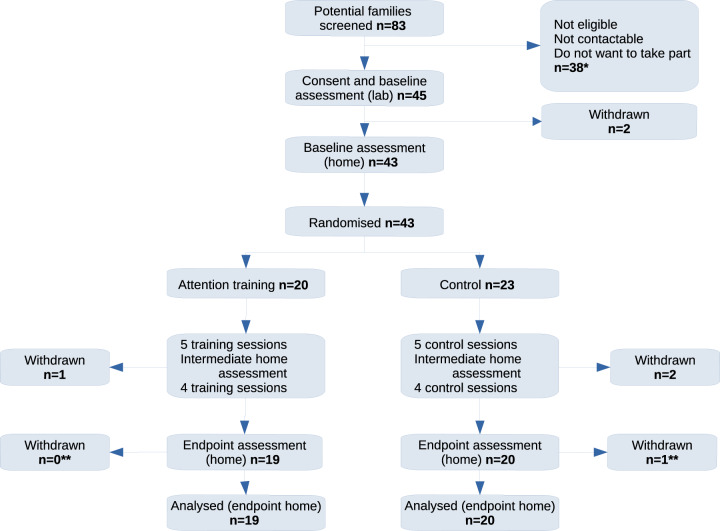
CONSORT Diagram. *In cases of twins (*N* = 2 families), one twin was randomised and included in analysis but both completed the full protocol. Due to the nature of the intervention, it would otherwise have been challenging to maintain parent blinding. **Presented Ns are for the endpoint assessment in the home only (see SM 1.7).

Forty-three 9- to 16- month-old infants with a first-degree relative (parent or older sibling) with a clinical or probable diagnosis of ADHD were randomised (see Fig. 1 and Table 1). Participants were recruited via a variety of routes including: NHS Trusts, recruitment databases at the Centre for Brain and Cognitive Development (Birkbeck, University of London), King’s College London, and at the University of Southampton (the South Hampshire ADHD Register and the Programme for Early Detection and Intervention), adult ADHD networks, ADHD support groups, radio adverts, newspaper articles, play centres, online forums, and charities.

**Table 1:** Study participant demographics and baseline characteristics.

	Controls (*n* = 23)	Training (*n* = 20)
Site London	14 (61%)	15 (75%)
Sex Female	11 (48%)	9 (45%)
Infants age (wks, mean (sd))	51.0 (6.6)	51.6 (6.9)
Gestational age (wks, mean (sd))	39.8 (1.3)	39.5 (1.5)
Annual family income (£1,000; median (IQR))^a^	50 (25-50)	57.5 (50-90)
Education of primary carer (tertiary or above)^b^	13 (57%)	14 (74%)

^a^Data are missing for 2 Control and 2 Training participants, ^b^Data are missing for 1 Training participant.

Inclusion criteria were (1) residence within a 2-hour travel distance from either London or Southampton, (2) at least one parent/caregiver who is fluent in English and, (3) a first-degree relative with a confirmed clinical diagnosis or research-probable diagnosis of ADHD. Where ADHD diagnosis had not yet been confirmed through clinical services, eligibility was confirmed using age appropriate standardised screening questionnaires to establish a probable research classification (see SM1.1, Table S1). Exclusion criteria were (1) serious medical or developmental conditions such as epilepsy, heart conditions, cerebral palsy, intellectual disability, (2) significant uncorrected vision or hearing problems, (3) significant prematurity (< 36 weeks), (4) genetic conditions (e.g. Down’s syndrome), (5) equipment unable to reliably track participant eyes during the baseline assessment after four attempts (*n* = 2).

### Randomisation and masking

#### Randomisation

Randomisation of individual children was completed after baseline and before the first training/control session using the King’s Clinical Trial Unit (KCTU) web-based service with minimisation over trial site and infant sex. Allocation was performed and automatically emailed to a trained researcher when they logged into the KCTU system.

#### Blinding

Researchers who administered the baseline and outcome assessments were blind to treatment condition. To maintain parent blindness, the number of home visits, contact with trainers, and set-up procedures were the same in the intervention and control groups. Data processing from control/intervention sessions and for primary outcome metrics were processed and extracted by researchers blind to group allocation. Analysis was completed using uninformative treatment labels. Subsequent to unblinding, recovery of data from three baseline eye-tracking sessions required analysis scripts to be rerun; interpretation of results did not change.

### Procedures

#### General design

Study flow is shown in Fig. 1. Briefly, after enrolment participants underwent baseline assessment first in a purpose-built laboratory at Birkbeck, University of London, and second at home. Infants were then randomised into the intervention and control groups and scheduled for weekly sessions of either the intervention or control package. Families were able to reschedule a session for up to two weeks after the target week, after which the session was skipped and the family moved on to the next session. An intermediate assessment was conducted after the 5th treatment or control session, and the outcome assessment after the intervention programme was completed (reaching the 9th training session, with a maximum of 3 of those sessions skipped; see SM1.2, Figure S1).

Each training/control session involved two trained researchers visiting the family’s home with the eye-tracking equipment (Tobii X2-60) (Figure S2 – S5). Adherence to the trial required a minimum of 6 training/control sessions, with a minimum average session duration of 5 minutes (>2SD below the mean of training durations in a pilot case series with typically developing infants, see SM1.3, Figure S6). Training fidelity was measured as the percentage of the time the child was looking at the screen that their eyes were detected during training visits.

#### Intervention

Six training games per session were run using in-house software (built in MATLAB R2016a with Psychophysics Toolbox3). Each training game was played for a maximum of 300 seconds, or until the infant became fidgety and disengaged for 20 seconds or more. If the infant had engaged with the game for fewer than 240 seconds (calculated by the software), the experimenters attempted to re-present the game again later in the training session. The software automatically recommended game order, which was pseudo-randomised across training visits (SM1.4). The games are described in full in Goodwin et al. [21] and SM1.4.

#### The Active Control Arm

Infants in the control group viewed infant-friendly but non-gaze contingent television clips under a similar procedure to the intervention and run using Task Engine 3 (built in MATLAB R2016a with Psychophysics Toolbox3 [22]). The set-up, procedures, duration and audio-visual style of the clips were designed to match the intervention condition as closely as possible (SM1.5).

### Outcomes

#### Primary outcome

The pre-specified primary outcome was a composite of measures (SM1.6) assessed at baseline, intermediate and endpoint. Designed to assess changes in infant attention control, the components were selected based on their responsiveness to attention training in previous studies and their widespread use in infant attention research [16]. Because we collected data in both the lab and the home, we pre-specified criteria in the Statistical Analysis Plan (SAP) (SM1.7) through which we determined whether lab or home data would form our primary assessments. Briefly, because 53% of the post-training lab-visits (< 75% cutoff) occurred within the stated 4 weeks of the last home training session (SM1.2) and >85% of infants had home data that met validity requirements for inclusion (see Table 2), then the home-based measures were used for the primary outcome.

1. *Sustained attention*: Infants watched five repetitions of each of two complex pictures each presented until the infant looked away for >1 second. The longest of the five individual looks towards each stimulus were averaged. A minimum of 4 ‘looks’ per infant (in either of the two blocks) was required.
2. *Disengagement*: Infants were presented with a minimum of 48 trials in which a central stimulus was followed by a peripheral stimulus that either did (Overlap) or did not (Baseline) overlap in time with the central stimulus. Disengagement scores were computed as the logged difference between mean saccadic reaction times in the Baseline and Overlap conditions. A minimum of 5 valid trials for the baseline and overlap conditions was required.
3. *Cognitive control*: Infants were presented with 18 trials in which after fixating a central fixation point, a pair of rectangles was presented. 2 seconds later or when a rectangle is fixated, a video appears on one side of the screen. After 9 trials, the location of the video was reversed. Cognitive control is measured as the percentage of trials in which infants correctly anticipate the location of the target stimulus across both phases. A minimum of 2 trials with anticipations (either correct or incorrect) per phase (learning and reversal) per block was required.

**Table 2:** Eye-tracker measurement of the three primary outcome components and overall looking to the screen (the only secondary outcome measured at all three timepoints).

	Control (*n* = 23)			Training (*n* = 20)		
	Baseline M (SD)	Intermediate M (SD)	Endpoint M (SD)	Baseline M (SD)	Intermediate M (SD)	Endpoint M (SD)
Disengagement	0.23 (0.15)^a^	0.18 (0.18)^d^	0.12 (0.09)^d^	0.21 (0.12)^a^	0.23 (0.10)^a^	0.17 (0.10)^b^
Sustained attention	0.29 (0.15)	0.37 (0.21)^c^	0.49 (0.22)^c^	0.25 (0.16)	0.38 (0.21)^a^	0.48 (0.25)^a^
Cognitive control	0.69 (0.16)^a^	0.74 (0.13)^d^	0.69 (0.14)^e^	0.73 (0.13)^a^	0.69 (0.16)^a^	0.69 (0.14)^b^
General inattentiveness (Secondary, % not looking to screen)	42.7 (13.5)	34.7 (14.8)^c^	32.5 (16.8)^c^	41.3 (11.4)	37.0 (12.5)^a^	31.2 (14.4)^a^

Data missing for: ^a^1 randomised participant, ^b^2 randomised participants, ^c^3 randomised participants, ^d^4 randomised participants, ^e^5 randomised participants.

For all tasks, the software computed an initial rapid estimate of validity in real time. If the validity criterion was not met at the end of the planned presentation trials, further ‘catch-up’ blocks were presented until the criterion was met. Of note, validity was then re-computed offline with more time-intensive but accurate methods and additional data could be considered invalid at this stage (see SM1.6).

#### Secondary outcomes

The following secondary outcomes were selected to assess whether changes in infant attention control were seen in more naturalistic contexts.

1. Parent-report measures of infant executive attention were assessed by the Infant Behavior Questionnaire-Revised shortened version [23]; specifically, the effortful control composite, activity level and duration of orienting scales.
2. Observational measures of infant attention to toys were assessed during the 3-minute task orientation episode from the Laboratory Temperament Assessment Battery, Lab-TAB [24]. Behavioural coding used an in-house coding scheme based on the Lab-TAB manual (SM1.8, Table S2).
3. Observational measures of infant social attention skills were assessed using the Early Social Communication Scales (ESCS; [25]). Responding to joint attention coded during the book task, and initiating joint attention were coded during the object spectacle and book task according to the ESCS manual (SM1.8, Table S3).
4. General inattentiveness was measured by proportion of missing eye tracking samples in the stimulus presentation area across the entire 20-minute eye-tracking battery.
5. Individual components of the primary composite were analysed separately.

#### Adverse effects

There have been no previous reports of any adverse effects of the training games. Though adverse effects were not expected based on previous studies using these training games, the monitoring of adverse events is important in early intervention studies [26]. Throughout the trial we monitored:

1. Infant sleep: Parents completed a Sleep Diary (SM1.9) the night before, and the night following each intervention session reporting the amount of sleep during the day and during the night (minutes), and the number of times the infant woke during the night.
2. Infant fussiness: Parents completed a fussiness scale (SM1.10) rated on a 1 to 5 scale for the afternoon of each intervention session, and for the afternoon of the previous day.

### Statistical analysis

The full Statistical Analysis Plan (SAP) was associated with Protocol version 3.0 and signed off by the DMEC and published online (ISRCTN37683928).

#### Sample size and power

Allowing for 10% attrition, the original proposed group size of 25 with baseline and single endpoint measures, correlated 0.6 over time, would have provided 82% power for a treatment effect size of 0.69 reported for cognitive control [16] (Stata sampsi procedure for analysis of covariance with two-tailed alpha=.05). The subsequent decision, following the pre-specified missing data rule, to use home measures as the primary outcome (see 2.5.1, Figure S7) allowed the inclusion of a second post-baseline mid-point measurement. Simulation of this three measurement design (correlated 0.6) for the analysis described below of the achieved sample size of 20 and 23, gave a non-central chi-square estimate of the power of 94% for the effect size of 0.69.

#### Statistical approach

To estimate the effect of treatment allocation following an intention to treat (ITT) approach, a multivariate analysis of covariance was fitted using maximum likelihood within the structural equation modelling (SEM) framework in Stata’s sem procedure. This allowed for an unstructured correlation matrix between the outcome components and was estimated by full maximum likelihood (Stata v15.1). Measures were assumed conditionally multivariate normal and missing values Missing-At-Random (MAR). Baseline and follow-up measures were treated as dependent variables with a design matrix of predictors that allowed factors for randomisation (sex and study site) and treatment group set to zero at baseline and a different error variance at baseline, midpoint, and endpoint with non-zero covariances over time and across indicators. Normal probability plots of residuals were examined. The model also provided correlation estimates of measurement reliability.

The above model provided six parameter estimates for treatment effect (intermediate and endpoint for each of cognitive control, sustained attention and disengagement components). Using the baseline standard deviation of each component, effect sizes were combined using the lincom post-estimation command (which takes the correlation among estimates into account), equally weighted across components but on the assumption that for each component the effect by mid-point would be half that achieved by endpoint. The 95% confidence interval for this combined ES was calculated using 1000 bootstrap samples.

The sign of the effect size for the disengagement component was reversed such that higher values denoted outcomes associated with older age, in line with the other two eye-tracking components. A Complier-Average-Causal Effect (CACE) analysis was conducted (SM1.11).

All secondary outcomes were continuously scored and analysed using a SEM setup that assumed bivariate normality of baseline and endpoint measures. All treatment group effects were adjusted for sex and study site except for the two Early Social Communication Scales (ESCS) that were further adjusted for age at administration.

For adverse events, averaged over sessions the before-minus-after difference scores were calculated for each participant for day and night sleep duration, and for fussiness. The group difference-score means were compared using t-tests.

## Results

### Demographics

After an extended period of 36 months of recruitment (11/2015–11/2018) 43 (Fig. 1) 9- to 16-month old infants with a first-degree relative with ADHD had been randomised to Control (*n* = 23) and Training (*n* = 20) groups (see Table 1 and SM2.1, Table S4).

### Follow-up, fidelity and adherence to training

Participant retention and follow-up collection of home data were good (SM2.2, Table S5). SM2.3 describes good comparability of duration and number of training and control visits. Of the 39 families randomised and who completed the trial, all met adherence criteria (min. 6 visits with 5 min training, Figure S8–S12); 19 of 20 in the Control group (95%) and 14 (73.7%) out of 19 participants in the Active Training group with a baseline assessment of the primary outcome met the CACE criteria (SM1.11) for good compliance with the trial programme. Eye tracking fidelity (% looking to screen while infant was attending during the training sessions) was M= 68.0 (SD 9.0; SM2.4, Figure S13).

### Primary eye-tracker assessed composite outcome and its components

**Fig. 2:**
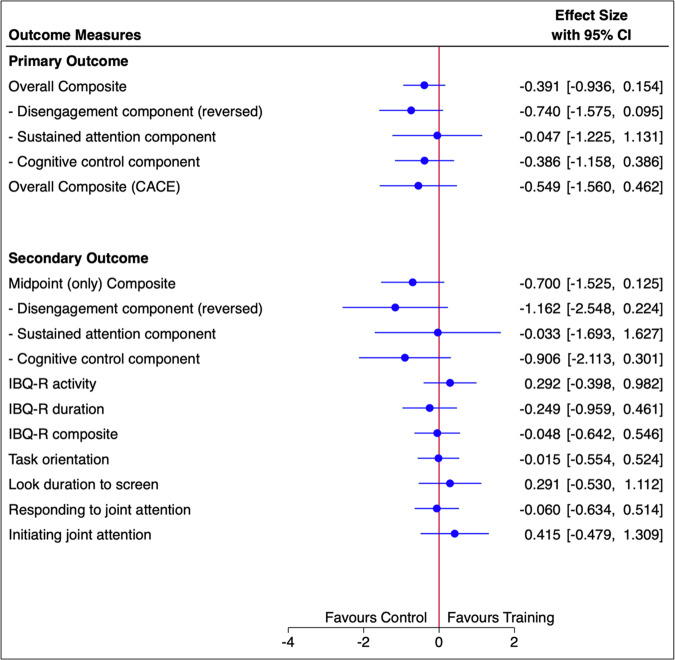
Forest plot of the effect sizes from the primary and secondary analyses. ITT estimates of Cohen’s d effect sizes based on baseline standard deviation except for Complier Average Causal Effect (CACE) estimator for overall composite. IBQ-R is the Infant Behaviour Questionnaire [23].

**Fig. 3:**
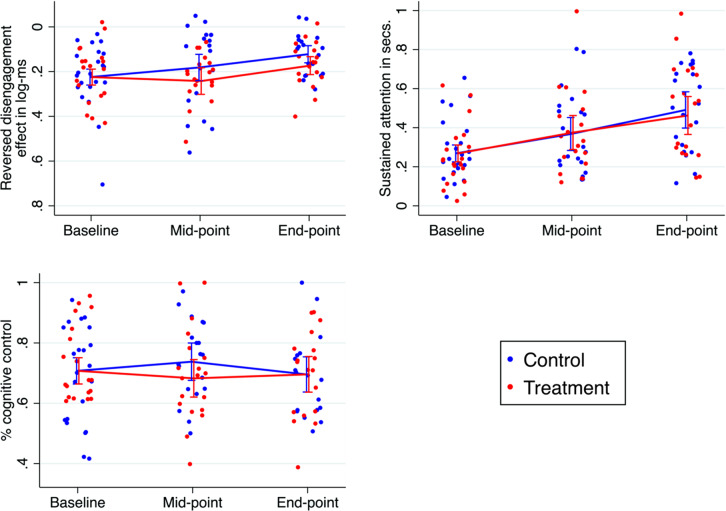
Plot of estimated group means for the three eye-tracking components. Analyses included a total of 43 participants. The Disengagement Effect has been reversed. Error bars are 95% confidence intervals around the average linear prediction.

Table 2 shows summary measures for the components of the primary outcome (see SM2.5 for reliability and covariation), and data completeness. Figure 2 summarizes the effect sizes for all outcomes and estimators. For the overall composite the treatment effect size combined over time was -0.39 (95% CI -0.94 to 0.15) and non-significant (p=0.16). Analysis of the individual components or of the components up to the intermediate test only (which corresponded more closely to the treatment durations of previous experiments) were also not significant (Fig. 3).

The CACE estimates the effect of a satisfactorily completed treatment schedule while accounting for the fact that those who comply may be systematically different from non-compliers. The CACE composite was not significant (effect size = −0.55; 95% CI −1.56 to 0.46, *p* = 0.29). The additional unplanned instrumental variable analysis of the effect of duration of Active Training was also non-significant (effect size −0.08, 95% CI −0.26 to 0.09, *p* = 0.35).

### Secondary naturalistic attention outcomes

Table 3 and Fig. 2 show the inconsistent and non-significant effect estimates for secondary outcomes, with nominal 95% confidence intervals and implied significance that do not account for the multiple comparisons.

**Table 3:** Secondary outcomes.

	Control (*n* = 23)		Training (*n* = 20)	
	T1 mean (SD)	T3 mean (SD)	T1 mean (SD)	T3 mean (SD)
Early Social Communication Scales				
Joint attention response	0.59 (0.26)^a^	0.74 (0.24)^d^	0.59 (0.29)	0.73 (0.19)^b^
Joint attention initiation	14.13 (8.06)	12.68 (11.26)^c^	13.4 (7.88)	16.56 (11.85)^b^
Infant Behaviour Questionnaire-Revised				
Activity level	4.49 (0.92)	4.14 (1.16)^e^	4.79 (1.03)^b^	4.63 (1.06)^c^
Orienting	3.39 (1.11)	3.66 (1.14)^e^	2.66 (0.83)^b^	3.29 (0.88)^c^
Effortful control	4.64 (0.54)	4.72 (0.61)^e^	4.56 (0.57)^b^	4.70 (0.73)^c^
Lab Tab				
Looking and manipulating (manlook)	56.1 (16.3)	56.84 (18.7)^c^	53.8 (19.2)	55.1 (15.4)^a^

Data missing for: ^a^1 randomised participant, ^b^2 randomised participants, ^c^4 randomised participants, ^d^5 randomised participants, ^e^6 randomised participants.

### Adverse events and effects

From the 17 controls and 15 training families who reported, hospital presentations were reported for three control (one night with high temperature, one ear-ache, and one dehydrated) and two training infants (one admitted for two nights with norovirus, and one for a barium meal bowel biopsy). The change in day and night sleep durations before and after a session showed no group difference (SM2.6). However, the training group were reported as showing a relatively greater increase in fussiness from before to after a session (M = 0.32, SD = 0.63) than the control group (M = −0.11, SD = 0.51) that was significant but small (.4 on a 5-point scale; *p* = 0.02). To assess whether there were any lasting effects on temperament, we conducted a DMC-approved non-prespecified examination of the IBQ-R Negativity composite (distress, fear, sadness, and falling reactivity) analysed in the same way as the other IBQ-R scales. No group difference was found (ES = 0.05, CI = −0.60 to 0.50; *p* = 0.862).

## Discussion

### Feasibility of pre-emptive intervention for ADHD

Intended as an infant-directed pre-emptive intervention for later ADHD, this trial is unique and innovative, and thus testing its feasibility was a primary aim. We undertook an extensive period of protocol development during an initial feasibility study (SM1.3). This resulted in a physical eye-tracking set-up that was acceptable to families and could be applied in the home to collect robust, high quality data. Indeed, the data we collected in the home was of sufficient quality to pass our strict quality assessment thresholds for selection as the primary outcome composite. We employed an active control arm that was matched in visit number, duration and protocol as closely as possible with the training arm, indicating that rigorous control designs are feasible in this population. Further, feedback questionnaires completed at the end of the trial indicated that families enjoyed participating in the study (SM2.7), with over 90% of respondents reporting that the visits were enjoyable or very enjoyable, 100% reporting that the sessions were of a suitable duration, and 100% reporting that the staff who visited their home were excellent. When asked what they found to be the most enjoyable part of the study, a key theme that emerged was seeing their infant’s behaviour. For instance, one parent reported that the most enjoyable part of the study was seeing their infant “learn how the sessions worked and remembering things”, while another reported that the most enjoyable part for them was “observing [their] baby’s reaction”. Another parent reported that their infant “really enjoyed the weekly visits” and that “watching his confidence grow was good.” This positive feedback provides strong encouragement for future home-based deployment of eye-tracking-based assessments and interventions for this population.

Despite these successes, we encountered a number of significant challenges. First, we targeted families for recruitment who had both a young infant, and also a parent or a child with ADHD. A proportion of families recruited were relatively poorly resourced (some annual incomes < £20,000). Around half of families additionally reported significant mental health or psychiatric conditions in a first degree relative. These factors can make recruitment and retention in trials difficult. Multiple recruitment methods had to be employed at both the London and Southampton sites. Additionally, matching rates of recruitment to home visit capacity (due to limitations of testing team time) was challenging. However, despite some cancelled appointments and re-scheduling, the protocol was successively administered to our cohort of 43 families. The total quantity of training (or control exposure) that infants received was greater than in previous studies (*M* = 224.57 minutes of total training, compared to 77 minutes in [16], SM2.3), albeit spread over a longer training period (93 days, compared to 16 days in [16], SM1.2). Given concerns that infants may, relative to adults and older children, show both greater plasticity and a faster rate at which training effects may potentially dissipate, the broader spread of training may have reduced efficacy in some cases. A slight increase in fussiness ratings from before to after each training session was noted in the intervention relative to the control group; however, this was small and did not result in longer term effects on negative emotionality measured at post-test.

Overall, we conclude that pre-emptive technology-based interventions directed at infants at elevated likelihood for ADHD are feasible. Further, these studies can and should apply rigorous RCT designs with appropriately designed control conditions, an approach that hitherto has been rarely taken with such populations [27]. As such, our study may provide a roadmap for future work. However, we did experience some challenges, particularly with participant recruitment. Once eligible families were identified, the conversion rate to enrolment was good (of the families contacted, only ~10% did not wish to participate). This indicates that the main barrier to recruitment was likely the restrictions of this specific trial (e.g. finding families who met the inclusion criteria for this particular trial), rather than reflecting low acceptability of the intervention approach more generally. Nevertheless, this highlights that further work is needed to reduce barriers so that a greater number of families can be reached in future trials of early interventions for ADHD. Current moves towards digital infrastructures such as trial platforms could be helpful in this regard [28, 29], potentially enabling more efficient recruitment strategies that better link researchers, clinicians and families. Community screening approaches (to identify parents or older siblings with elevated ADHD symptoms) could also help to reach families who may be missed by other recruitment strategies, such as those who do not have a clinical diagnosis of ADHD. The current study was also restricted to families who lived within 2 hours of one of the study site centres. Shifting to an in-home parent-administered set-up may help to improve recruitment in future home-based eye tracking studies, by removing such geographical barriers and allowing for greater numbers of families to be reached.

### Outcome of the training

This trial yielded null results for both primary and secondary outcome measures. Some of the effect sizes we observed (around 0.39) indicate that attention skills around the end of the first year may be malleable, and that variations on the procedures we used, perhaps delivered with greater intensity or over a longer time period, may still be worth pursuing. One area for future exploration concerns the degree of contingency needed for optimal learning. For the training tasks, our fidelity measures showed that, on average, the eye tracker detected the infant’s gaze 68% of the time that they were looking to the screen. Such tracking validity is common even in lab-based developmental eye tracking [30] but here was exacerbated by home testing. This means that sometimes the games reacted as though the infant was not looking when they were. It may be that future improvements in eye tracking hardware will allow more efficient implementation of the games and therefore a more effective training paradigm. Further, it may be that the density or number of sessions should be increased for this group of infants relative to typically developing children. However, we note that the primary outcome effect size is comparable to other early intervention studies [27], but is not in the predicted direction. This highlights that in order to design effective early interventions for ADHD we need greater understanding of the nature of the developing attention system in infancy; how individual differences in attention in infancy relate to longer-term behavioural outcomes; and the mechanisms through which the developing attention system can be strengthened. Our study was designed based on the most replicated infant attention training paradigm currently available [16], but did not show robust effects. Our work indicates that such studies are now feasible, but that significant effort is required to generate robust and generalisable early intervention paradigms in this area.

The present RCT was designed with later emerging ADHD traits in mind, and the possibility of pre-empting some attention difficulties before they become embedded during the course of development. While the study is not sufficiently powered to address later clinical diagnosis of ADHD, scheduled assessments of the participants at 24 and 36 months will allow us to measure any longer term effects on traits, or on sub-clinical attention difficulties. Indeed, a recent pre-emptive trial of a parent-mediated intervention with infants with older siblings with autism showed a significant effect on symptoms over time despite no immediate post-training effect [31]. Combining the present design with an adaptation of parent-training approaches designed for older children [32] such that training can be administered with greater density and intensity (an immersion in an attention-supporting context) may also be a critical step forward in this field. However, it may be that the present trial is consistent with recent meta-analyses in indicating that cognitive training-style approaches to ADHD treatment are largely ineffective [12].

### Limitations

A limitation of the study is the sample size. While the target sample size for this study was 50 infants, 43 infants were randomised. The trial was powered on the basis of findings from previous experimental studies that provided the best available evidence for the likely effect size (e.g. [16]). Our confidence intervals do not entirely rule out smaller but clinically significant beneficial effects. However, the largest effect in our study (−0.39) was not in the predicted direction. Infant gaze is likely influenced by a range of environmental (such as socioeconomic status, SES) [33, 34], and genetic [35] factors. Our sample was not large enough to investigate subgroups (e.g. based on SES), nor was this a primary aim of the trial. Including larger, diverse samples in future home-based eye tracking studies could help to dissect the role that individual differences in environmental and genetic factors may have on measures of infant visual attention.

Another limitation of the study is that thus far, the infants have not been followed up into toddlerhood and preschool. In this study, we targeted infant attention skills. Infant attention skills in both screen-based [36] and naturalistic [37] contexts have been shown to relate to later ADHD traits. However, there is some recent evidence that difficulties may be more apparent in other neurocognitive domains in the early development of ADHD, such as activity levels [38]. Moreover, the early development of attention involves a complex interplay both with other cognitive domains, such as learning and memory [39, 40], as well as environmental factors such as parenting behaviours [41] and living environments [42]. Thus, targeting attention skills alone may not have a significant effect on later ADHD traits, and perhaps a more multi-modal approach to intervention is required. However, though differences in infant attention may not distinguish children at an elevated likelihood for ADHD [38], executive attention skills may potentially act as a neurocognitive modifier, changing the developmental trajectory of associations between early differences in brain development and later behaviours related to the ADHD phenotype [43]. Follow-up of this cohort at 24 and 36 months of age will allow us to investigate potential longer term effects of the training on preschool ADHD traits, or sub-clinical attention difficulties.

A further limitation of the study is that the majority of families included in the trial were white (93% of mothers, and 90% of fathers, SM2.1). Under-recruitment of families from certain ethnic backgrounds may partly reflect a broader issue in the diagnostic process for ADHD, where ethnic and racial disparities have been shown [44, 45]. Future studies investigating early interventions for ADHD should focus on ensuring a more representative sample. Moving away from typical ad hoc recruitment processes towards digital tools for trial recruitment may increase trial visibility and potentially improve inclusivity and participation of under-represented groups [29].

## Conclusions

In conclusion, our study provides an important demonstration of a rigorously designed pre-emptive RCT at home for infants at elevated likelihood for ADHD. While the present study did not yield positive results, the feasibility of the intervention approach will provide an important impetus for paediatricians, ADHD researchers and psychologists to turn their attention to very early intervention in this population.

## Data sharing statement

Individual participant data that underlie the results reported in this article, after de-identification (text, tables, figures, and appendices) will be made available through the data sharing procedures of the BASIS network (basisnetwork.org). This includes presentation of a methodologically sound proposal with appropriate data security and ethical clearances to comply with the terms of the original collection. The study protocol and statistical analysis plan are freely available through the ISCRTN page and in [21]. Data will be available immediately following publication with no end date.

## Supplementary information

Supplementary Materials
