# Supplementary material for: INTERSTAARS: Attention training for infants with elevated likelihood of developing ADHD: A proof-of-concept randomised controlled trial
Source: Transl Psychiatry. 2021 Dec 20;11:644. doi: 10.1038/s41398-021-01698-9 (PMC8688472; doi:10.1038/s41398-021-01698-9)
Supplement: Supplementary file 1 — Supplementary Materials [file 41398_2021_1698_MOESM1_ESM.docx]

# Supplementary Materials

[1. Supplementary Methods 2](#_Toc82594011)

[1.1. Further information about diagnostic status 2](#_Toc82594012)

[1.2. Spacing of training/control sessions 4](#_Toc82594013)

[1.3. Development of home-based set-up 6](#_Toc82594014)

[1.4. Further details on training tasks 11](#_Toc82594015)

[1.5. Control condition 15](#_Toc82594016)

[1.6. Primary outcome eyetracking task battery 15](#_Toc82594017)

[1.7. Rules for selection of lab or home data (reproduced from the SAP: ISRCTN37683928) 17](#_Toc82594018)

[1.8. Coding scheme for behavioural secondary outcome measures 18](#_Toc82594019)

[1.9. Infant sleep 21](#_Toc82594020)

[1.10. Infant fussiness 21](#_Toc82594021)

[1.11. CACE analysis 21](#_Toc82594022)

[2. Supplementary Results 22](#_Toc82594023)

[2.1. Further participant characteristics 22](#_Toc82594024)

[2.2. Data completeness for key measures 26](#_Toc82594025)

[2.3. Infant engagement. 28](#_Toc82594026)

[2.4. Eyetracker fidelity during training 32](#_Toc82594027)

[2.5. Covariation between composite components 33](#_Toc82594028)

[2.6. Adverse events 33](#_Toc82594029)

[2.7. Parent feedback 34](#_Toc82594030)

[3. References 35](#_Toc82594031)

## Supplementary Methods

### Further information about diagnostic status

We defined the presence of ADHD as a community clinical diagnosis of ADHD or a probable research diagnosis of ADHD in a first-degree relative. Information about first-degree relative diagnostic status was ascertained through a number of methods. Before families enrolled in the study, a telephone screening form was used to determine the presence of an existing clinical diagnosis of ADHD in a first-degree relative (parent or older sibling, Table S1). This was confirmed by interview with parents at their infant’s first visit. A proportion of children/parents had suspected ADHD, but this had not yet been confirmed by clinical services (N = 15, Table S1). For those who reported suspected ADHD, screening questionnaires were used to examine the probable existence of ADHD. Inclusion decisions were reviewed by the trial management and oversight teams. Our categorisation protocol is similar to that adopted by other labs using the prospective longitudinal study model in infants at elevated likelihood of ADHD (see Miller et al., 2020).

For siblings (6 years or older), a shortened adapted version of the Conners 3 [1] was used. Behaviours that parents reported as occurring either “often” or “frequently” were scored. All included children met a minimum threshold for inclusion of i) 6 ADHD symptoms on either the hyperactivity/impulsivity scale (consisting of item numbers: 3, 43, 45[54]*, 61, 69[99]*, 71, 93, 98, 104) or the inattention scale (consisting of item numbers: 2, 28, 35, 47, 68[79]*, 84, 95, 97, 101), and ii) a positive score on the impairment scale (at least 2 out of 3 impairment items, consisting of item numbers: 106, 107, 108). ^1^

For siblings (aged less than 6 years), a shortened adapted version of the Conners Early Childhood [2] form was used. Behaviours that parents reported as occurring either “often” or “frequently” were scored. All included children met a minimum threshold for inclusion of i) 9 ADHD symptoms on the inattention/hyperactivity scale (consisting of item numbers: B8, B12, B22, B34, B42, B47, B49, B55, B65, B72, B74), and ii) a positive score on the impairment scale (at least 2 out of 3 impairment items, consisting of item numbers: IM1, IM2, IM3).

For parents, a shortened adapted version of the Conners Adults ADHD Rating Scale (CAARS) [3], either self or observer report. Behaviours that parents reported as occurring either “often” or “frequently” were scored. All included parents met a minimum threshold for inclusion of 5 ADHD symptoms on either the hyperactivity/impulsivity scale (consisting of item numbers: 2, 4, 6, 8, 16, 18, 22, 25, 27) or the inattention scale (consisting of item numbers: 1, 9, 13, 14, 19, 21, 26, 29, 30). Of note, the adult version of the Conners does not include impairment questions.

*Indicates that these two items were collapsed into a single question in the adapted screening form.

**Table S1.** Categorisation of the ADHD probands for randomised participants (N = 43)

| **Proband with ADHD** | |
| --- | --- |
| Parent | 24 |
| Older sibling | 18 |
| Parent & Older sibling | 1 |
| **Diagnostic status** | |
| Clinical diagnosis | 28 |
| Probable research diagnosis (Conners-3) | 5 |
| Probable research diagnosis (Conners-EC) | 3 |
| Probable research diagnosis (CAARS) | 7 |

^1^Note, one older sibling scored 6 for inattention items and 6 for hyperactivity/impulsivity items, but only scored 1 for impairment. Upon reviewing the case with senior clinicians (TC, PB), this family was included in the study. It was considered that the impairment questions were an underestimate because the child was receiving very high levels of support both at home and at school (and thus impairments were mitigated and less clear).

### Spacing of training/control sessions

According to the planned design (see main text section 2.4.1), testing sessions were scheduled to be weekly, with families able to reschedule for up to two weeks after the target week. If a family had not rescheduled at any point, the intermediate assessment session would have taken place 42 days (6 weeks) after the home baseline visit, and the endpoint assessment 77 days (11 weeks) after the home baseline visit. 34% of intermediate home visits and 13% of post-test home visit took place within +/- 2 days of these time windows (Figure S1). The mean (std) number of days between the Baseline and Intermediate assessments was 56.95 (14.8) for the trained group and 49.6 (9.1) for the control group. The mean (std) number of days between the Baseline and Endpoint assessments was 94.65 (16.9) for the trained group and 93.1 (10.9) for the control group, approximately 2.5 weeks later than if no rescheduling had occurred.

The between-participants variability in the spacing of the training/control visits was higher than encountered in previous studies. For comparison, Figure S1 (right) shows the same figure for a 2011 study [5]. The planned testing schedule for this 2011 study was for 5 visits spread over 15 days, with the post assessment (equivalent to the intermediate visit in this study) scheduled for day 15. The mean (std) days between visit 1 and the post assessment was 15.8 (1.6) for the trained group and 15.5 (1.5) for the control group. 93% of visits took place within +/- 2 days of the planned post assessment date. However, the intervention reported in 2011 was planned over a much shorter period (two weeks vs 11 weeks), with fewer visits (5 vs 11) and with typically developing infants rather than infants with older siblings or parents with ADHD.

Note that although the planned schedule was to implement visits weekly, we anticipated that given the length of this study in comparison to previous studies (e.g. Wass et al. 2011 [5]), that there would be some deviance from this. Therefore, we implemented a “two week rule” whereby a visit could be rescheduled within two weeks of the planned visit. If a visit could not be rescheduled within two weeks of the planned schedule then this visit was cancelled, e.g. if training visit 4 could not be implemented within 2 weeks of the planned schedule then the next training visit for this infant would be training visit 5.


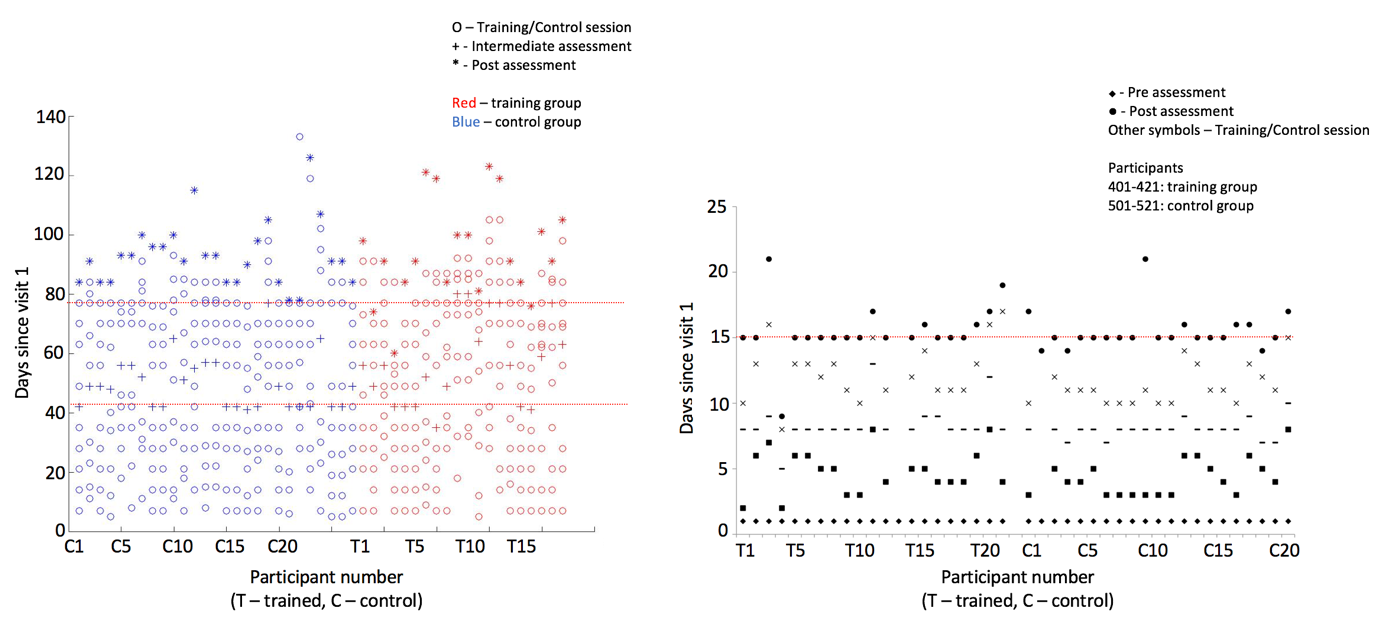


*Figure S1 Left: spacing of training/control sessions, and intermediate/endpoint assessments, in days since Visit 1 (the baseline assessment). Red shows the intervention group; blue, the control group. Lines show when the intermediate assessment (day 42) and endpoint assessment (day 77) would have been carried out if no visits were rescheduled. Right: equivalent figure showing the spacing of training control sessions, and post assessment from the 2011 study [5]. Line shows the planned date for the post-test assessment (day 15).*

### Development of home-based set-up

During each session, infants were seated on their parent’s lap and surrounded by a photography tent to prevent visual distraction; stimuli were presented on a Dell 19-inch monitor screen, with a screen resolution of 1024×768 pixels (Figure S2). This set-up was refined through an extensive piloting process described below. Assessments were video-taped. During both training sessions and outcome assessments parents were instructed to comfort their infant when necessary, but not to direct their attention.

**
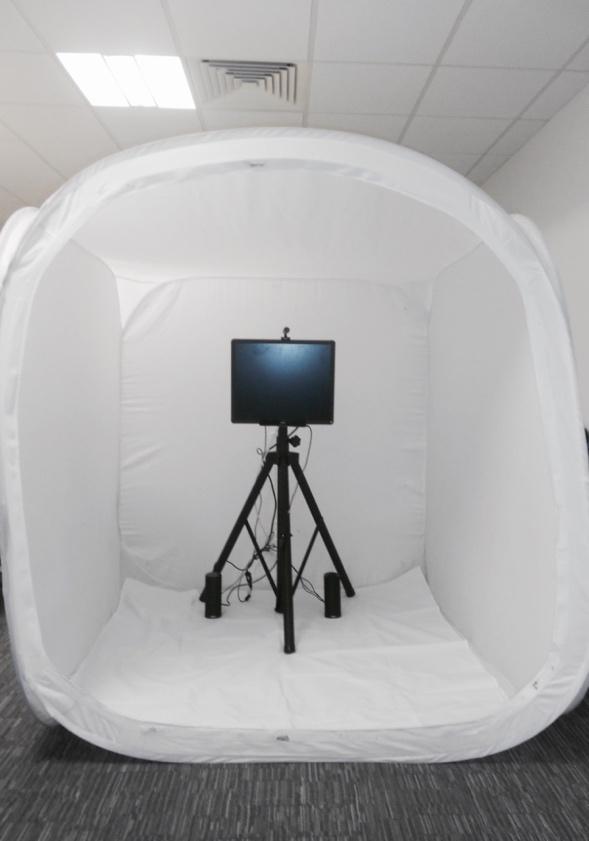

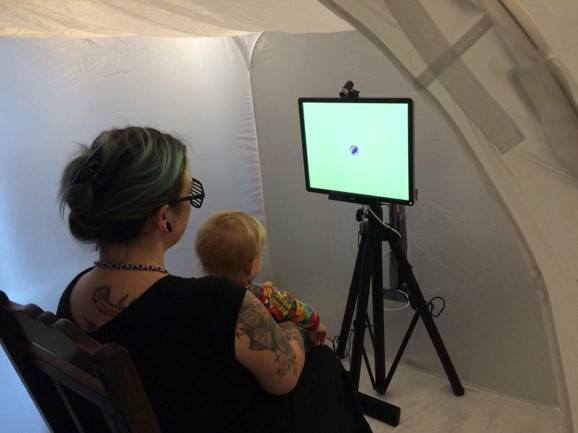
**

*Figure S2. Final set up used in the INTERSTAARS trial to collect eye-tracking data in the home. This is based on Setup D (see below), which was optimal in terms of data quality metrics but included a more portable tent that required minimal time to set up in families’ homes. Consent was obtained to publish these images.*

Before the INTERSTAARS trial began, two pilot studies were conducted to assess the feasibility and acceptability of the study protocol.

#### Study 1

In Study 1, a pilot study was conducted to optimise the feasibility of using eye-tracking in the home with infants. Data quality metrics using an eye-tracking set up that could be administered in families’ homes, and a portable Tobii X2-60 eye-tracker, were obtained from a sample of typically developing infants (N = 36; 18M, 18F; *M* age = 361.32 days). Based on data quality metrics (Figures S3-5) and experimenter observations of infant behaviour and practicalities, adaptations were made to the eye-tracking set up throughout the pilot study. This resulted in four different set ups being trialled (A-D) using a ~20-minute battery of eye-tracking tasks. Data quality was compared to data collected in the laboratory with a Tobii TX300 (Figures S3-S5) with infants of a similar age (*M* age = 320.67 days), and a comparable eye-tracking battery. Based on the results of this piloting, the final home set up used in the INTERSTAARS trial is shown in Figure S2.

Four data quality metrics were examined. These were:

1. % No Eyes detected: This was calculated as the number of samples where the eye-tracker was not detecting either of the infant’s eyes divided by the total number of samples during the battery.


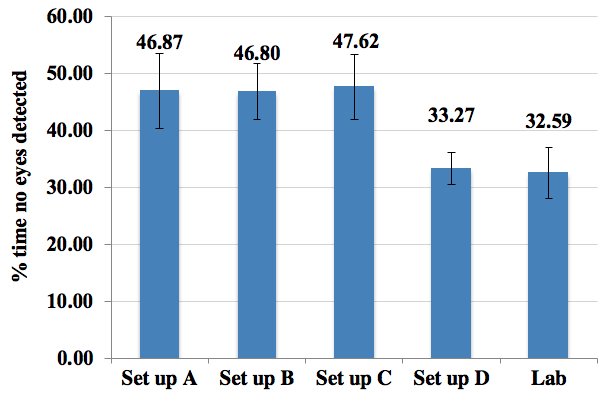


*Figure S3. Mean percentage of time the eyetracker could not detect the infant’s eyes across portable set ups A-D and data collected in the laboratory. In terms of data quality, a lower percentage is better. Error bars are SE of the mean.*

1. Fixation RMS (Root Mean Square): Fixation RMS was used to measure precision. The root-mean-square difference between each consecutive pair of gaze samples was calculated in the X and Y axes. Resulting RMS values for each axis were then averaged.

**
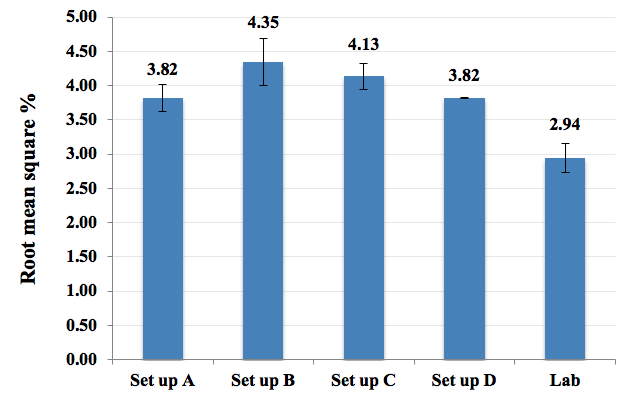
**

*Figure S4. Mean fixation root mean square (RMS) across portable set ups A-D and data collected in the laboratory. This value represents the variability in the spatial location of recorded gaze within a period of fixation at one location. In terms of data quality, a low fixation RMS value is better. Error bars are SE of the mean.*

1. Flicker ratio: Flicker ratio reflects the proportion of consecutive samples that recorded the same value for eye detection (yes or no), and was calculated as the proportion of consecutive sample pairs that were the same divided by the overall number of sample pairs. Poor eyetracker contact often results in eye detection varying from sample to sample. High flicker ratios represent better quality data.

**
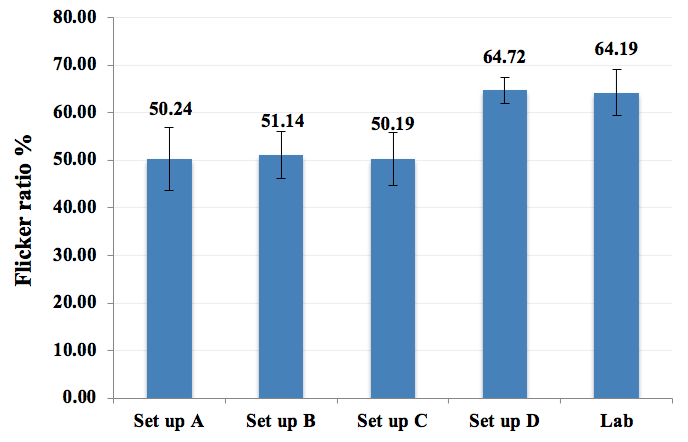
**

*Figure S5. Mean flicker ratio across portable set ups A-D and data collected in the laboratory. In terms of data quality, a higher flicker ratio is better. Errors bars are SE of the mean.*

#### Study 2

In Study 2, a small case series study was conducted (N=4 infants; 8.5 – 17 months of age), that implemented the full intervention protocol. Of note, an additional participant initially consented to the case series study but dropped out before any data was collected (researchers were unable to contact the family after three consecutive attempts). As no data was collected for this participant, and the research team were not able to contact the family, it was not possible to include this participant in further analysis.

The case series was conducted to assess whether parents found the protocol acceptable. To assess acceptability we examined attrition rates, and conducted qualitative interviews with parents at the end of the study. We found that implementing the attention training in the home, over an 11-week period, was acceptable to families. The mean number of training sessions completed was 10 out of a possible 11 sessions. The maximum number of training sessions was 11 (three out of four participants received 11 training sessions). The minimum number of training sessions was 8 (one participant received 8 training sessions). The training times for case series participants across all visits are shown below in Figure S6. In INTERSTAARS, 9 training visits were implemented. The mean training times for case series participants across the first 9 visits was 14.97 minutes (SD = 4.19).

*Figure S6. Infant training times (in minutes) across weekly training visits for case series participants*

Qualitative interviews with the primary caregiver indicated that implementing the attention training in the home was acceptable to the families who took part in the case series study. A common theme across the interviews was that all parents liked that the research was conducted in their home. Two parents attributed this to the practicalities and inconvenience associated with travelling to and from a lab with a young infant each week, with one parent saying that they would not have participated in the study had it not been conducted in the home, and another saying that if it had not been conducted at home “it would turn from effectively a 45 minute exercise to a ... 2.5 hour, 3 hour exercise”. One parent also commented that the flexibility of the researchers worked well, and that there does need to be some flexibility in terms of dates and times for home visits each week, when the programme runs for a long length of time. In terms of future families taking part, families referred to how their own  personal home set up worked well. All parents mentioned that certain circumstances (e.g. being able to organise care for older siblings during the attention training sessions, having a table and chair at home, or being a working parent) might vary across families and make it easier or more difficult to administer the home-based attention training.

### Further details on training tasks

The attention training programme used gaze-contingent animations that operated via eye-tracking technology to target attention control. Infants viewed animated games on a computer screen in which where they looked on the screen determines what they see. The games were intended to be attractive and enjoyable for infants. The games were adaptive, so that as the infant performed better, the levels increased, and the games became more challenging. The original training programme featured four games [5]. In order to maintain engagement over the longer training phase planned for INTERSTAARS (nine versus four training sessions), additional versions of the original games were added to the training programme, closely based on the original set but with different surface features. Together with one new game, they were designed to train attention control across mixed cognitive domains that include sustained attention, working memory, visual search and inhibitory control. As described in the main text, six tasks were presented at each training session, following the following procedure: Sessions 1-6: Flyme, Stars, Suspects, Butterfly, Windows, Puzzle Memory. Sessions 6-9: Flyme, Stars, Suspects, Platform, Tausendfuss, Three Maids. Presentation order within each session was pseudo-randomised with the only criterion being that the same training task was not presented first as had been presented first at the previous session.

#### FlyMe:

In the FlyMe game, the infant is presented with a cartoon character. When the infant looks directly to the cartoon character, an animation shows the character ‘flying’ upwards and scrolling distractors (e.g. clouds, aircraft, stars) scroll down from the top of the screen. If the child looks to any of these distractors they disappear and the animation freezes. When the child looks back to the main character, the ‘flying’ animation restarts. The salience of the distractors varies contingent on performance.

#### Stars:

In the Stars game, the infant must search for the correct target within a complex visual scene. The target (a cartoon character in a brightly coloured star) is presented alongside eight distractors (e.g. planets, clouds). The infant must look to the target within a time limit. If they correctly locate the target they receive an animated reward sequence. As the infant progresses successfully through trials, the levels become more difficult by increasing the salience of the distractors.

#### Suspects:

In the Suspects game, a target (a colourful cartoon elephant) is presented alongside a distractor. If the infant fixates on the target elephant within a certain time period then they receive an animated reward sequence. A larger number of distractors are presented contingent on performance. Every 12 trials, the target switches: where previously the child has received a reward for looking to the elephant, they now start to receive a reward for looking to a chicken. The target changes periodically between the elephant and the chicken, changing every 12 trials.

#### Butterfly:

In the Butterfly game, the infant is presented with a cartoon butterfly on the computer screen. When the infant fixates on the butterfly it flies across the screen, from left to right. As it flies, various distractors (clouds, trees, houses) scroll from left to right. If the child looks to any of the moving (and therefore highly likely to be visually salient) distractors, they disappear and only the butterfly remains on-screen. The child has to learn that the only way to progress on the task is to look to the butterfly and inhibit the prepotent urge to look to distractors. The salience of the distractors increases contingent with performance, i.e. the better performing infants are trained with more frequent and more salient distractors. Two of the other games included in the training programme use the same training paradigm as Butterfly, with new graphics and sound files.

#### Windows:

In the Windows game, the infant is presented with two windows on the left and right of the computer screen. A cartoon earwig is presented in one window, together with an attention-getter (a red circle) and an audio sound effect to draw the infant’s attention to the earwig. Once the child has looked to the earwig, it disappears, and the infant is presented with a fixation point (a cartoon flower) in the centre of the screen. When the infant fixates on the flower it rotates until a delay period has elapsed, and then disappears. If the infant then looks to the correct window (where the earwig was previously displayed) they receive a reward sequence showing the earwig flying off the screen, accompanied by sound effects. As the infant progresses through the difficulty levels, the delay period becomes longer, and the number of windows increases. Another game included in the training programme uses the same training paradigm as Windows, but with new graphics and sound files.

#### Puzzle Memory:

In the Puzzle Memory game, a cartoon character is presented in one of two locations. Following an animation, the character disappears and a fixation point is presented elsewhere on the screen. If the child subsequently looks back to the location where the character disappeared and maintains their gaze there, the character reappears. A second character is then presented in the second location, followed by the same sequence (character disappears, fixation point is presented). However throughout this sequence, the previously found character remains on screen. During the response window, the infant has to use the stored memory to maintain their gaze on the location where the new character has disappeared, and inhibit the urge to look to the previously found character, which is more immediately salient. At higher difficulty levels, the number of locations increases. Another game included in the training programme uses the same training paradigm as Puzzle Memory but with new graphics and sound files.

#### Tausendfuss:

This game is identical in structure to Puzzle Memory, but with alternative graphics, sound files and reward animations.

#### Platform:

In the Platform game, the child views a screen like a 2-D scrolling computer game. A cartoon character is shown in the left hand corner of the screen. When the child looks directly at the character, then an animation shows the character walking and distractors (clouds, other platforms, other characters) scroll in from the right hand side of the screen. If the child looks to any of these distractors, the animation stops. When the child looks back to the character, then the animation re-commences. The difficulty (salience of the distractors) varies contingent on performance.

#### Three Maids:

In Three Maids, the child sees a character appearing, then disappearing into one of several hiding locations (e.g. plant pots). A distractor (a spider appearing at the top of the screen) is then presented. If the child looks back to the hiding location after the distractor disappears, and maintains their gaze there for a short time, an animation shows the character re-appearing and a reward sequence is triggered. The number of hiding locations and the length of the distractor vary contingent on performance.

### Control condition

Each television clip was scaled to full screen, with the aspect ratio maintained. Clips were played with their original audio. Frame rates for the clips range from 23 to 30 frames per second. Individual clips from the same television programme were combined into a “theme”. There are nine themes in total, mirroring the nine training games in the intervention. The same fixed rules used to deliver the intervention were also used to administer the control condition. Six themes were played per session. The MATLAB scripts automatically recommend which theme the researcher should play next, according to an order that was pseudo-randomised across sessions. Each theme was played for a maximum of 300 seconds, or until the infant becomes fidgety and has not been engaged for 20 seconds or more. The same objective measures used to assess the delivery of the intervention, e.g. session duration, number of themes played, were also collected for the control arm.

### Primary outcome eyetracking task battery

#### Cognitive control, adapted from [4, 5]

At the beginning of each trial, the infant is presented with a fixation point in the centre of the screen. Once the infant looks to the fixation point, an audio reward is presented, followed by a visual reward (a short animated clip), which is presented on either the left or right of the screen. The visual reward is presented on 1 side for 9 consecutive trials (pre-switch) before switching to the other side for the subsequent 9 trials (post-switch). Anticipatory saccades are coded based on the child’s looking behaviour during the anticipatory window (between the start of the auditory reward and the start of the visual reward). The dependent variable is the percentage of trials in which infants make a correct anticipatory saccade towards the location of the target stimuli in the pre- and post-switch phases.

#### Sustained attention, adapted from [5]

The infant is presented with two ‘interesting’ (complex, detailed) and two ‘boring’ (noncomplex) static stimuli. For each stimulus, the experimenter records the length of the first 5 of the infant’s looks towards the stimulus presentation area by depressing a button while the infant is looking. To qualify as a look the infant must visually engage with the stimulus for at least 1 s. To terminate the look, the infant must disengage from the stimulus for at least 1 s. The longest of the first 5 looks is termed the peak look duration. The dependent variable is the peak look duration averaged across the two blocks of the interesting stimulus presentation.

#### Gap-overlap, adapted from [6]

The infant is presented with a stimulus in the centre of the screen (central stimulus, CS). Once the infant fixates on this central stimulus, a peripheral stimulus (PS) appears on the left or right of the screen. When the infant moves their gaze from the central to the peripheral stimulus they receive an audiovisual reward. There are three conditions in this task: baseline, overlap and gap. In the baseline condition, the central stimulus disappears at the same time that the peripheral stimulus appears. In the gap condition, there is a 200-ms gap between the removal of the central stimulus and the appearance of the peripheral stimulus. In the overlap condition, the central stimulus remains on the screen after the peripheral stimulus appears. The dependent variable is the saccadic reaction time (ms) to move the eyes from the central to the peripheral stimulus in the overlap minus baseline condition.

**Online validation:** The task was presented in blocks of 12 trials. Each trial was coded as valid or invalid after presentation. Valid trials were those in which: 1) gaze remained on the CS until PS onset; 2) gaze arrived at the PS within 1200ms of PS onset; 3) gaze did not arrive in less than 200ms; 4) gaze did not go to the opposite side to the PS (indicative of anticipation). If after the fourth block (48 trials) fewer than 12 valid trials per condition (baseline, gap, overlap) had been acquired, a fifth block was run (total 60 trials).

**Offline validation:** During analysis, each trial of the gap was re-inspected using inhouse software (TaskEngine 3), and re-coded for validity. This allowed a more fine-grained check of the data than during online validation, where time was constrained by the need to present the next trial. Valid trials were those in which: 1) gaze remained on the CS until PS onset, with no contiguous runs of missing data greater than 200ms; 2) gaze remained on the PS after PS onset, with no runs of missing data greater than 100ms; 3) gaze was on the CS within +/-50ms of PS onset, with no runs of missing data greater than 50ms; 4) gaze did not go to the opposite side to the PS; 5) saccadic reaction time was not less than 200ms or greater than 1200ms. Final analysis is based on offline validation codes.

### Rules for selection of lab or home data (reproduced from the SAP: [ISRCTN37683928](http://www.isrctn.com/ISRCTN37683928))

As described in the protocol section 4.7.1, treatment blind analysis will be carried out to assess quality of data collected during testing sessions. Therefore, we will use a home-lab composite primary outcome measure score *unless* the following conditions are not met for at least 75% of the sample:

1. Cognitive control task: a minimum of 2 trials with anticipations (either correct or incorrect) per phase (learning and reversal) per block.

2. Gap-overlap task: a minimum of 5 valid trials per critical condition (baseline and overlap).

3. Sustained attention: a minimum of 4 'looks' per infant during interesting phase (in either one of the two blocks administered).

4. Measurement Lag: Time between last home session and post-training lab-visit less than 4 weeks.

If these conditions are not met for 75% of the sample, then we will use *either* the home measures only (if 1, 2 and 3 are met for 75% of infants for the home battery, but 4 is not met) or the lab measures only, (if 1, 2 and 3 are not met for the home battery but 4 is met) to calculate the final composite score.

If this is not possible (e.g. 1, 2, 3 and 4 are not met for the home battery) then we will select either the lab or the home composite on the basis of which battery has the greater proportion of infants who meet criteria 1, 2 and 3.

#### Catch-up scripts:

After each pre/post session, the data was examined to determine the number of valid trials for each task (gap: 5; cognitive control: 2 valid antisaccades per side, per block; habituation: 4 looks in at least “interesting” block). In cases where any task did not have enough valid trials per condition, a catch-up session was run before the first training/control session, prior to randomisation. In catch-up sessions, only those tasks with low valid trials per condition were run again.

### Coding scheme for behavioural secondary outcome measures

#### Measures of general attention: Blocks task

In this task, the infant sat on their parent’s lap at a table, or in a high chair and was given a set of 12 blocks of different colours and shapes to play with for 3 minutes. The below variables were coded offline continuously at 25 frames per second using Mangold INTERACT version 15. All coders were blind to group randomisation.

| **Table S2: Codes used for the Block task** |
| --- |
| Manipulation of block  *Proportion of time spent manipulating the blocks. Manipulation was defined as a frame in which the child was touching and/or holding the blocks. Manipulation does not include throwing the blocks off the table or mouthing the blocks.* |
| Looking at blocks  *Proportion of time spent looking at the blocks. Looking at the blocks was defined as the child visually fixating one or more of the blocks.* |
| Manlook*  *Manlook was the proportion of time the child spent looking at and manipulating the blocks. This was identified through the overlap between the looking and manipulating codes (calculated within Mangold Interact).* |

*Variable selected for secondary outcome analyses ([Interstaars Statistical Analysis Plan](http://www.isrctn.com/editorial/retrieveFile/6ae446f2-50c7-4904-90ef-728502c4c61b/30831)). Inter-rater reliability for “Manlook” (single measures, absolute agreement; 100% videos double coded): ICC baseline = .908; ICC endpoint = .956.

#### Social attention task (the Early Social Communication Scales/ESCS)

In this task, infants sat on their parent’s lap at a table, with the researcher sitting opposite and facing the infant. In advance of the home visit, parents were asked whether they had a table that could be used for this task. In cases where families did not have a table in their home, the researcher made their best judgement on where to set up this task within the home environment (following discussion with the parent).

The object spectacle, social interaction, gaze following, and book presentation tasks of the ESCS (Mundy et al. 2003) were administered. The variables initiating joint attention (IJA) and responding to joint attention (RJA) were selected for secondary outcome analyses ([Interstaars Statistical Analysis Plan](http://www.isrctn.com/editorial/retrieveFile/6ae446f2-50c7-4904-90ef-728502c4c61b/30831)). Counts of IJA and RJA behaviours were coded offline (see Table S3 below).

The primary coder was blind to group randomisation. For IJA, a second blind coder double-coded 73% of videos for inter-rater reliability (single measures absolute agreement, ICC IJA baseline = .89, ICC IJA endpoint = .89). For RJA, a second (not blind) coder double-coded 100% of videos for interrater reliability (single measures, absolute agreement, ICC RJA baseline = .79, ICC RJA endpoint= .86). Note for both IJA and RJA, only the blind primary coder’s data was used for analyses.

**Table S3.** Codes used for secondary outcome analyses from the ESCS

| **Code** | **Description** |
| --- | --- |
| **Responding to joint attention** | Joint attention during the book task (defined as per the ESCS manual, Mundy et al. 2003), excluding trials where the infant was already looking at the book at the beginning of the trial, and including only immediate looks (total immediate looks/number of trials). |
| **Initiating joint attention** | Joint Attention coded during book and object spectacle tasks; total of the two lower (Eye Contact and Alternates) and two higher (Points and Show) forms as specified on page 50 of the ESCS manual (Mundy et al. 2003). |

### Infant sleep

Parents were given a sleep diary to complete for the day and night before, and the day and night of, each training / control home visit (adapted from the Oxford Sleep Questionnaire). The diary asked parents to record what i) time they put their infant down for a sleep, ii) time their infant slept from, iii) time their infant slept until, and iv) where their infant slept. Daytime and night sleeps were recorded separately. Parents were also asked whether there was anything unusual that may have disrupted their infant’s sleep for that 24 hour period, such as illness.

### Infant fussiness

Parents were asked to rate how fussy their infant was on a 1 - 5 scale (where 1 = not at all fussy, 5 = very fussy) for the afternoon before and the afternoon of each training or control visit.

### CACE analysis

The Complier-Average-Causal Effect (CACE) was estimated using a two-stage (instrumental variable - IV) estimator in which the treatment group in the Training arm was replaced by the predicted probability of compliance from the first stage probit model for the binary compliance indicator. Briefly, non-compliance for the purposes of the CACE analysis was prespecified in the SAP as failing two of the following three criteria: a) more than 21 days elapsed between the last training/control visit and the endpoint or intermediate test; b) more than 114 days elapsed between the baseline and endpoint; or more than 70 days between the baseline and intermediate assessments OR between the intermediate and endpoint assessments. A similar two-stage IV approach, with a first stage predicting duration of training, estimated the effects of a continuous duration of training measure; this was not prespecified in the SAP.

## Supplementary Results

### Further participant characteristics

**Table S4.** Study participant demographics and baseline characteristics

|  |  | | | **Control**  **(N = 23)** | **Training**  **(N = 20)** | **Total**  **(N = 43)** |
| --- | --- | --- | --- | --- | --- | --- |
| ***Infant Baseline Demographics*** | | | | | | |
| Site | | London *n* | | 14 | 15 | 29 (67%) |
|  |  | Southampton *n* | | 9 | 5 | 14 (33%) |
|  | | Missing *n* | | 0 | 0 | 0 |
| Sex | | Female *n* | | 11 | 9 | 20 (47%) |
|  |  | Male *n* | | 12 | 11 | 23 (53%) |
|  |  | Missing *n* | | 0 | 0 | 0 |
| Gestational age (weeks) | | Mean (SD) | | 39.8 (1.3) | 39.5 (1.5) | 39.6 (1.4) |
|  | | Missing *n* | | 0 | 0 | 0 |
| Infant age (weeks) | | Mean (SD) | | 51.0 (6.6) | 51.6 (6.9) | 51.3 (6.7) |
|  |  | Missing *n* | | 0 | 0 | 0 |
| Mullen Scales of Early Learning Composite | | Mean (SD)  Range | | 80.6 (12.00)  61 – 104 | 83.1 (9.8)  61 – 99 | 81.7 (10.9)  61 – 104 |
|  |  | Missing *n* | | 1 | 1 | 2 |
| ***Parent-report measures of infant characteristics at baseline*** | | | | | | |
| SSQ  Average duration of night sleeps in the past week  Average duration of day sleeps in the past week  Average number of night wakings in the past week | | | Mean (SD)  Missing *n*  Mean (SD)  Missing *n*  Mean (SD)  Missing *n* | 555.3 (166.8)  3  111.2 (46.1)  2  2.2 (2.2)  3 | 620.6 (149.3)  4  127.1 (44.4)  3  1.6 (1.4)  3 | 584.3 (160.4)  7  118.3 (45.4)  5  1.9 (1.89)  6 |
| VABS-II  Adaptive Behaviour Scale (Composite score) | | | Mean (SD) | 102.4 (11.8) | 96.8 (13.7) | 99.6 (12.9) |
|  |  |  | Missing *n* | 2 | 1 | 3 |
| Minutes per day of screen exposure (TV) | | | Mean (SD) | 23.7 (18.1) | 34.6 (31.7) | 28.9 (25.7) |
|  | | | Missing *n* | 2 | 1 | 3 |
| ***Family Baseline Demographics*** | | | | | | |
| Age (years) biological mother | | | Mean (SD) | 33.1 (6.1)  0 | 34.1 (3.8)  1 | 33.6 (5.2)  1 |
|  |  |  | Missing *n* |  |  |  |
| Age (years) biological father | | | Mean (SD) | 34.3 (6.3)  0 | 36.3 (4.6)  1 | 35.2 (5.7)  1 |
|  |  |  | Missing *n* |  |  |  |
| Ethnicity of biological mother^a^ | | | Asian %  Black %  White %  Mixed %  Other %  Missing *n* |  |  | 0  0  92.9  7.1  0  1 |
| Ethnicity of biological father^a^ | | | Asian %  Black %  White %  Mixed %  Other %  Missing *n* |  |  | 0  2.4  90.5  4.8  2.4  1 |
| Country of birth biological mother^a^ | | | UK *n*  Australia *n*  Brazil *n*  France *n*  Israel (*N*)  S. Africa (*N*)  Thailand (*N*)  USA (*N*)  Missing *n* |  |  | 32  1  1  1  1  2  1  3  1 |
| Country of birth biological father^a^ | | | UK *n*  Brazil *n*  Denmark *n*  Guyana *n*  S. Africa *n*  USA *n*  Zimbabwe *n*  Missing *n* |  |  | 36  1  1  1  1  1  1  1 |
| Education level of primary caregiver | | | Below tertiary  N (%)  Tertiary  N (%)  Missing *n* | 10 (44%)  13 (57%)  0 | 5 (26%)  14 (74%)  1 | 15 (36%)  27 (64%)  1 |
| First language of primary caregiver | | | English primary  *n* (%)  Language that is not English  *n* (%)  Two first languages, including English  *n* (%)  Missing *n* | 20 (87%)  1 (4%)  2 (9%)  0 | 18 (95%)  1 (5%)  0 (0%)  1 | 38 (91%)  2 (5%)  2 (5%)  1 |
| Proportion of language that infant hears in English | | | 100% English  *n*  70%-99% English *n*  50–69% English *n*  Less than 50% English *n*  Missing *n* | 18  4  1  0  0 | 16  1  2  0  1 | 34  5  3  0  1 |
| Infant has first degree relative with:^c^ | | | ASD  *n* (%)  Depression and/or anxiety  *n* (%)  Psychiatric other^b^  *n* (%)  Genetic syndrome  *n* (%)  Missing *n* | 10  (46%)  12  (55%)  1  (5%)  0  (0%)  1 | 3  (16%)  8  (42%)  0  (0%)  1  (5%)  1 | 13  (32%)  20  (49%)  1  (2%)  1  (2%)  2 |
| Infant has second degree relative with:^c^ | | | ASD  *n* (%)  Depression and/or anxiety  *n* (%)  Psychiatric other^b^  *n* (%)  Genetic syndrome  *n* (%)  Missing *n* | 4  (18%)  5  (23%)  5  (23%)  2  (9%)  1 | 2  (11%)  8  (42%)  5  (26%)  0  (0%)  1 | 6  (15%)  13  (32%)  10  (24%)  2  (5%)  2 |
| Infant has other relative (ie relative that is not first or second degree) with:^c^ | | | ASD  *n* (%)  Depression and/or anxiety  *n* (%)  Psychiatric other^b^  *n* (%)  Genetic syndrome  *n* (%)  Missing *n* | 1  (5%)  0  (0%)  1  (5%)  1  (5%)  1 | 3  (16%)  0  (0%)  0  (0%)  1  (5%)  1 | 4  (10%)  0  (0%)  1  (2%)  2  (5%)  2 |

SSQ = Sleep and Settle Questionnaire; VABS II = Vineland Adaptive Behaviour Scales Version 2 Parent Report

^a^Variables that could identify a single case at the individual level are not shown summarised by group (training and control)

^b^Includes: Schizophrenia, bipolar disorder, manic depression, been admitted to hospital for a psychiatric condition

^c^Infants are only presented once per condition category (under the highest degree relative). For example, if an infant had a first and second degree relative with ASD, they are presented under “Infant has first degree relative with ASD”.

### Data completeness for key measures

Data was collected in both the home and the lab. The [Interstaars Statistical Analysis Plan](http://www.isrctn.com/editorial/retrieveFile/6ae446f2-50c7-4904-90ef-728502c4c61b/30831) (page 14) pre-specified a set of criteria for selecting the primary outcome from i) a lab-home composite, ii) home measures only iii) lab measures only. Following these pre-specified steps, the home measures were selected for the primary outcome (Figure S7).


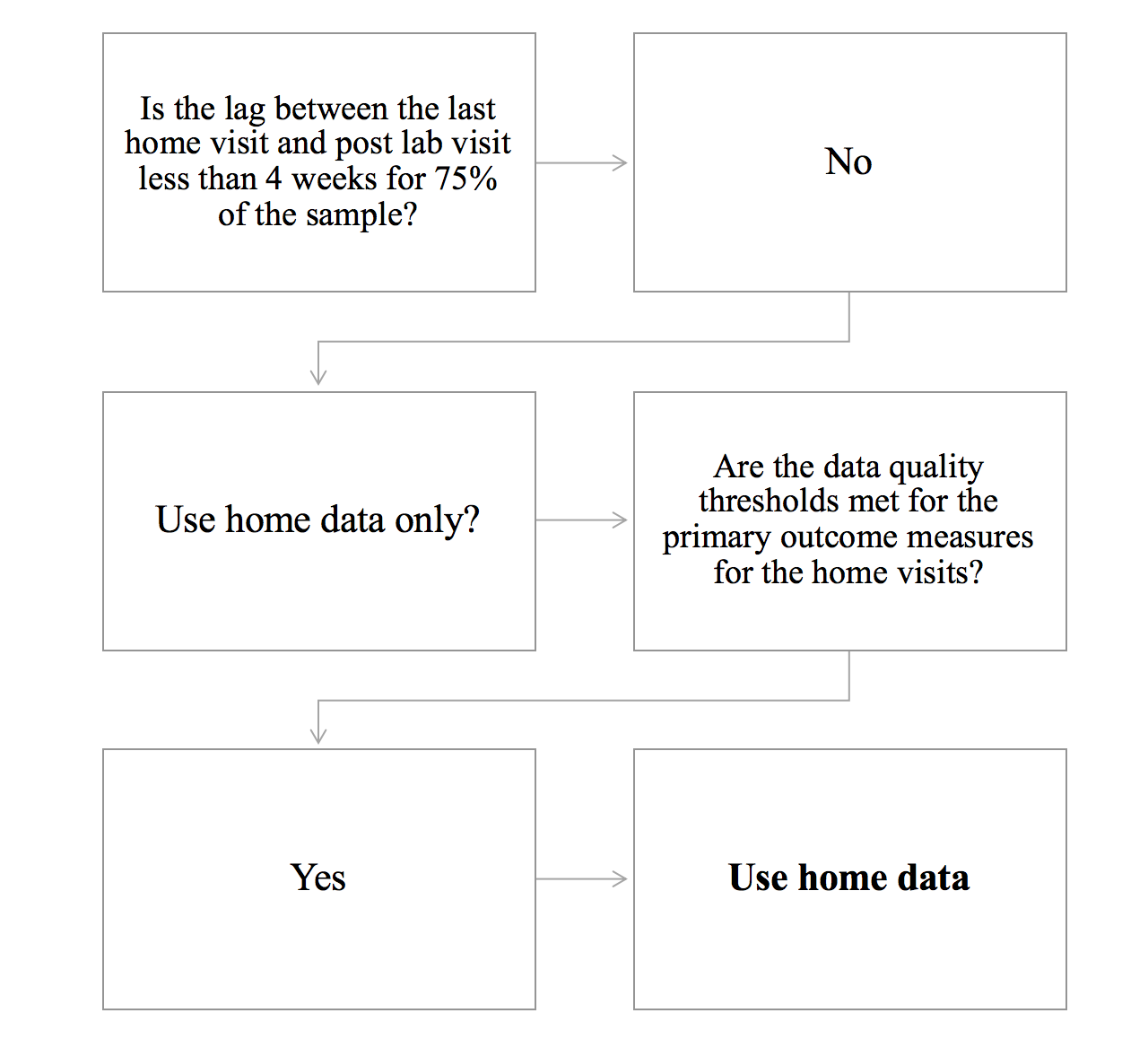


*Figure S7. Decision tree for selecting the primary outcome measure*

As shown in above in Figure S7, the home data was selected for analysis following a pre-specified procedure for outcome selection. Table S5 shows data completeness for the primary and secondary outcomes completed in the home.

**Table S5.** Data completeness for primary and secondary outcomes

| BASELINE | | |
| --- | --- | --- |
| **Primary Outcome (Eye Tracking)** |  |  |
|  | %Completed | %Completed that met data quality criteria |
| Sustained attention | 100 | 100 |
| Disengagement | 100 | 95.3 |
| Cognitive control | 100 | 95.3 |
| **Secondary Outcome (Behavioural)** |  |  |
|  | %Completed | %Completed that met data quality criteria |
| LabTAB Task orientation | 100 | 100 |
| ESCS Initiating Joint Attention | 100 | 100 |
| ESCS Responding to Joint Attention | 100 | 97.6 |
| IBQ | 95.3 | 100 |
| ENDPOINT | | |
| **Primary Outcome (Eye Tracking)** |  |  |
|  | %Completed | %Completed that met data quality criteria |
| Sustained attention | 90.7 | 100 |
| Disengagement | 88.4 | 97.4 |
| Cognitive control | 88.4 | 94.7 |
| **Secondary Outcome (Behavioural)** |  |  |
|  | %Completed | %Completed that met data quality criteria |
| LabTAB Task orientation | 90.7 | 97.4 |
| ESCS Initiating Joint Attention | 86.1 | 100 |
| ESCS Responding to Joint Attention | 86.1 | 97.3 |
| IBQ | 76.7 | 100 |

Of note, data completeness for primary outcome measures was also higher in the home than in the lab. Data completeness for the measures completed in the lab are shown below.

% Completed for lab eye tracking measures

|  | ***Baseline*** | ***Endpoint*** |
| --- | --- | --- |
|  |  |  |
| Sustained attention | 93 | 79.1 |
| Disengagement | 95.3 | 79.1 |
| Cognitive control | 95.3 | 79.1 |

### Infant engagement.

#### Intervention

As described in the main text, each training game was played for a maximum of 300 seconds, or until the infant became fidgety. Figure S8 (left) shows the average presentation time for each game; since, on occasion, each training task was presented more than once at a given visit, the longest instance of each training task at a given training session was calculated for this measure. Differences between games are a consequence of the fact that some of the training tasks were less engaging, and so infants tended to become fidgety more quickly. Figure S8 (right) shows the number of times that each training task was presented across the whole training battery. Figure S9 shows the total amount of training (in minutes) that each infant received. Figure S10 shows the length of every individual training session that was administered over the course of the study. This indicates that the protocol for training task presentation was complied with.


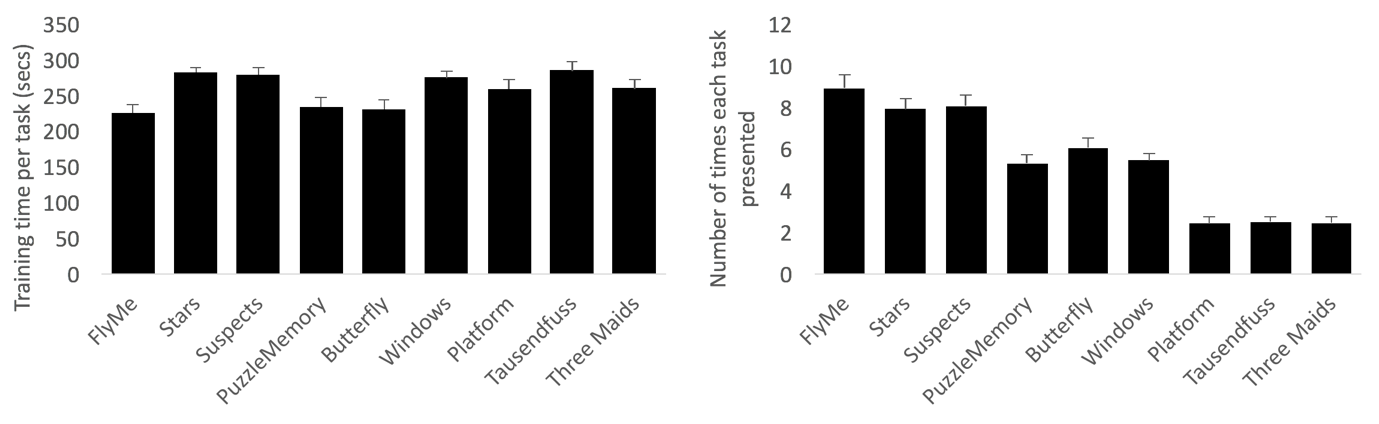


*Figure S8. Left: average training time (in seconds) for each occasion that each training task was presented. Right: number of times that each training task was presented.*

*Figure S9. The total amount of training (in minutes) that each infant received (M = 224.57 minutes).* *This excludes one infant who dropped out of the study.*

*
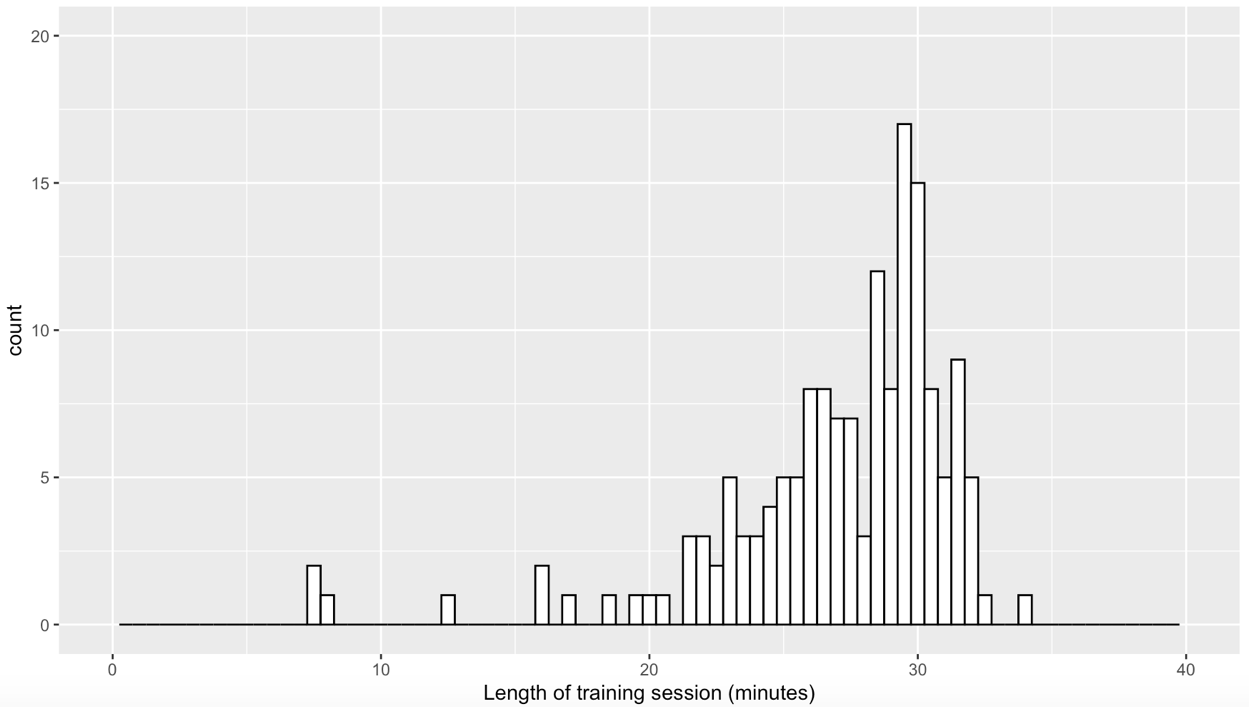
*

*Figure S10. Histogram with the length of each individual training session conducted during the study on the x-axis (rounded to the nearest whole minute) and the number of training sessions per session length on the y-axis.*

#### Control

The same procedure described for the training games above was used to administer the control condition. Clips from the same televisions programme were combined into a ‘theme’. There were nine control themes (similar to there being nine training games). Each control theme was played for a maximum of 300 seconds, or until the infant became fidgety. Figure S11 Left Panel shows the number of times each control theme was presented and Figure S12 Left Panel shows the average proportion looking time to each control video (with Figure S11 and 12 Right Panel presenting comparative data from the training arm).

­

*Figure S11. Left: number of times that each control video was presented. Right: number of times that each training task was presented.*

*Figure S12. Average proportion of looking per individual participant across all training visits.*

### Eyetracker fidelity during training

The operation of the gaze-contingent games depends on the degree to which the games are responsive to the infant. In turn, this is dependent on the degree to which the infant’s gaze is captured by the eyetracker when they are looking at the screen. When infants move a lot, or are seated in a sub-optimal position, the eyetracker may fail to detect their eyes, and the games will respond as though the infants are not looking at them when they in fact are. In order to assess fidelity to the training task, three measures were calculated (see main text – section 2.4.1). First, we recorded the proportion of valid eyetracker data obtained while the training games were running (Figure S13 left). The mean (SD) value was 0.61 (0.11). Second, we recorded the proportion of infant viewing observed while the training games were running, as coded live by the experimenter using a key-press. The mean (SD) value was 0.81 (0.07). Third, we recorded the proportion fidelity, indexed as the correspondence between hand-coding of when the infants were attending to the screen, and the successful detection of gaze from the eyetracker. The mean (std) value was 0.68 (0.09). Of note, this value can be affected by both poor quality tracking or inaccurate hand coding of attention.

*
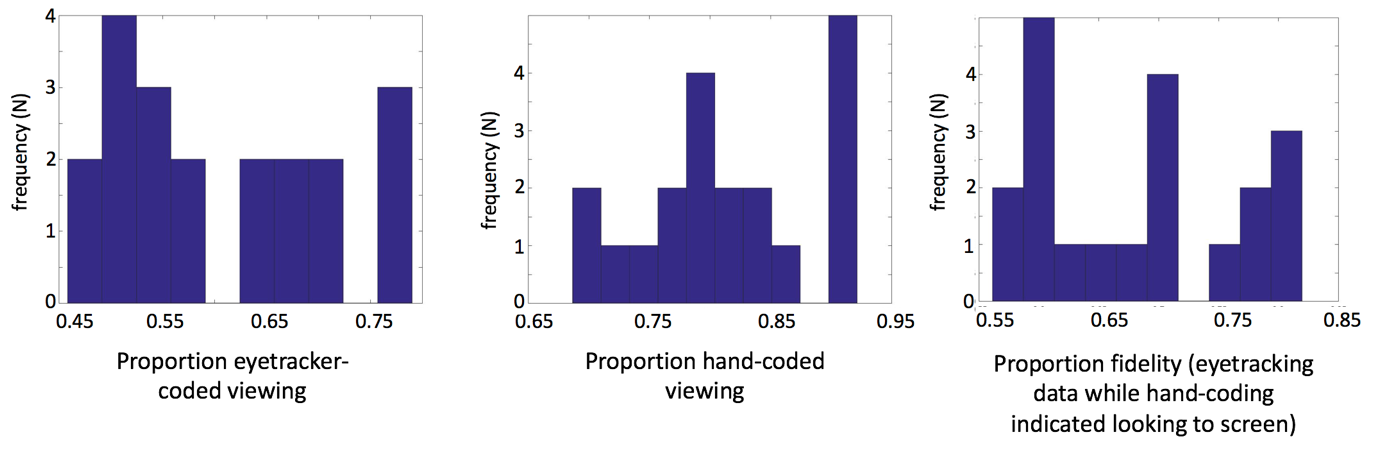
*

*Figure S13. Left: histogram showing the proportion of valid eyetracker data recorded while the training games were running (only trained group is shown). Centre: histogram showing the proportion of infant viewing observed while the training games were running, as coded live by the experimenter using a key-press. Right: histogram showing the proportion fidelity (correspondence between hand-coding of when the infants were attending to the screen, and the successful detection of gaze from the eyetracker.*

### Covariation between composite components

Model-based reliability estimates from baseline to endpoint (~3 months apart) were 0.48 for the disengagement component, 0.35 for the sustained attention component, and 0.39 for the cognitive control component. The baseline means of the three eye-tracking components were similar for the two groups with small between-component correlations (r= 0.06 between disengagement and sustained attention, -0.20 between disengagement and cognitive control, and 0.26 between sustained attention and cognitive control).

### Adverse events

|  | **Controls (n=23)** | **Training (n=20)** | **Group diff t-test**  **p value** |
| --- | --- | --- | --- |
| **Infant fussiness:**  **Day-of-session minus day-before mean difference (SD)** | -0.11 (0.51)^b^ | 0.32 (0.63)^a^ | 0.02 |
| **Sleep Diary:**  **Day-of-session minus day-before mean difference (SD)** | -8.73 (23.0) ^b^ | -1.91 (26.1) ^a^ | 0.39 |
| **Sleep Diary:**  **Night-of-session minus night-before mean difference (SD)** | 1.29 (36.6) ^b^ | -6.45 (29.9) ^a^ | 0.47 |

Data missing for: ^a^ 1 randomised participant, ^b^ 2 randomised participants.

### Parent feedback

Anonymous feedback questionnaires were given to the parent/caregiver after their infant had completed the final home visit. Of those who completed the feedback questionnaire (N = 15), responses are shown in the table below.

| **Question** | **Parent feedback (N = 15)** |
| --- | --- |
| *Please rate how satisfied you were with the duration of the home-based visits* | 1 – Too short  2 -  3 – Perfect length (100% of respondents)  4 -  5 – Too long |
| *Please rate how satisfied you were with the number of the home-based visits* | 1 – Too many  2 – (6.7% of respondents)  3 – Perfect number (80% of respondents)  4 – (13.3% of respondents)  5 – Too few |
| *Please rate how satisfied you were with the staff that visited you in your home* | 1 – Poor  2 –  3 – Average  4 –  5- Excellent (100% of respondents) |
| *Please rate how enjoyable you and your baby found the sessions that were carried out in your home* | 1 – Very enjoyable (53.3% of respondents)  2 – (40% of respondents)  3 – Fairly enjoyable  4 – (6.7% of respondents)  5 – Not enjoyable |

Furthermore, 100% of respondents reported that they would recommend the study to other families.

We also asked parents “what was the most enjoyable aspect of the home visits?” A key theme was seeing their baby’s behaviour, particularly how their baby interacted with the research team. Example quotes from parents to this question are shown below:

“seeing baby learn how the sessions worked and remembering things”

“baby really enjoyed the weekly visits - watching his confidence grow was good”

“baby sleep well after. Comfortable. Fun to see her behaviour”

“observing baby's reaction”

“got on very well with the staff and I think they made the experience very easy and pleasant.”

“definitely having the researchers visit every week watching her interaction with them”

“she liked interacting with the team and enjoyed lots of the videos”

“she loved the tent and chatting to new people”

## References

1. Conners CK. Conners 3rd edition. Toronto: Multi-Health Systems Inc; 2008.
2. Conners CK. Conners Early Childhood Manual. NY: Multi-Health Systems Inc; 2009.
3. Conners CK., Erdhardt, D., Sparrow, E. Conners Adults ADHD Ratings Scales (CAARS). NY: Multi-Health Systems Inc; 1999.
4. Kovács, Á. M., & Mehler, J. (2009). Cognitive gains in 7-month-old bilingual infants. *Proceedings of the National Academy of Sciences*, *106*(16), 6556-6560.
5. Wass, S., Porayska-Pomsta, K., & Johnson, M. H. (2011). Training attentional control in infancy. *Current Biology*, *21*(18), 1543-1547.
6. Elsabbagh, M., Volein, A., Holmboe, K., Tucker, L., Csibra, G., Baron‐Cohen, S., ... & Johnson, M. H. (2009). Visual orienting in the early broader autism phenotype: disengagement and facilitation. *Journal of Child Psychology and Psychiatry*, *50*(5), 637-642.
